# Targeting macrophages in atherosclerosis using nanocarriers loaded with liver X receptor agonists: A narrow review

**DOI:** 10.3389/fmolb.2023.1147699

**Published:** 2023-03-02

**Authors:** Tong-Mei Yang, Miao Miao, Wen-Qian Yu, Xue Wang, Fang-Jie Xia, Yan-Jie Li, Shou-Dong Guo

**Affiliations:** Institute of Lipid Metabolism and Atherosclerosis, Innovative Drug Research Centre, School of Pharmacy, Weifang Medical University, Weifang, China

**Keywords:** Liver X receptor agonists, macrophages, atherosclerosis, nanodrug delivery system, cholesterol homeostasis

## Abstract

Macrophages are involved in the whole process of atherosclerosis, which is characterized by accumulation of lipid and inflammation. Presently, clinically used lipid-lowering drugs cannot completely retard the progress of atherosclerosis. Liver X receptor (LXR) plays a key role in regulation of lipid metabolism and inflammation. Accumulating evidence have demonstrated that synthetic LXR agonists can significantly retard the development of atherosclerosis. However, these agonists induce sever hypertriglyceridemia and liver steatosis. These side effects have greatly limited their potential application for therapy of atherosclerosis. The rapid development of drug delivery system makes it possible to delivery interested drugs to special organs or cells using nanocarriers. Macrophages express various receptors which can recognize and ingest specially modified nanocarriers loaded with LXR agonists. In the past decades, a great progress has been made in this field. These macrophage-targeted nanocarriers loaded with LXR agonists are found to decrease atherosclerosis by reducing cholesterol accumulation and inflammatory reactions. Of important, these nanocarriers can alleviate side effects of LXR agonists. In this article, we briefly review the roles of macrophages in atherosclerosis, mechanisms of action of LXR agonists, and focus on the advances of macrophage-targeted nanocarriers loaded with LXR agonists. This work may promote the potential clinical application of these nanocarriers.

## Introduction

It is known that the onset and progression of atherosclerosis are associated with elevated low-density lipoprotein (LDL) particles as well as raised TG-rich lipoproteins (Vekic et al., 2022; Zhang B. H et al., 2022). Although the first line lipid-lowering drugs, statins, are capable of lowering LDL cholesterol (LDL-C) and suppress inflammation, these drugs can only reduce ∼30% cardiovascular disease (CVD) events. Furthermore, clinical studies have found various side effects of statin therapy (Qiao et al., 2022; Zhang W et al., 2022). A promising alternative to statin therapy is the inhibition of the proprotein convertase subtilisin/kexin-9 type, thereby increasing the uptake of circulating LDL particles by the liver (Qiao et al., 2022). However, they are not suitable for primary prevention because they are not cost-effectiveness. Additionally, the clinically used TG-lowering drugs, fibrates, are still inconclusive for therapy of atherosclerotic CVD (Zhang B. H et al., 2022; Jin et al., 2023). Currently, some novel therapeutic strategies have been proposed, such as gene-guided therapy, anti-inflammatory therapy, regulation of immunity, endothelial function, and gut microbiota (Björkegren and Lusis, 2022; Chan and Ramji, 2022). Presently, there is no effective strategies to successfully reduce or eliminate atherosclerotic plaques that have formed.

Atherosclerosis is characterized by the formation of atherosclerotic plaques, which are composed of lipids, necrotic nuclei, calcified areas, inflammatory cells, endothelial cells, immune cells, and foam cells derived from macrophages (Liu et al., 2021). Macrophages play an important role in innate and adaptive immunity as well in tissue repair and internal homeostasis. On the one hand, macrophages differentiate into different subtypes and exert distinct functions in the microenvironment of atherosclerotic plaques (Lin et al., 2021). On the other hand, macrophages play a pivotal role in regulation of lipid homeostasis within atherosclerotic plaques (Lin et al., 2021; Björkegren and Lusis, 2022). However, the normal physiological function of macrophages is disturbed in atherosclerosis. For instance, macrophages are the main inflammatory cells in atherosclerotic lesions, playing a leading role in the formation, development, and regression of atherosclerotic plaques (Björkegren and Lusis, 2022). Targeting the recruitment and activation of normal macrophages or regulation of dysfunctional macrophages are promising therapeutic strategies.

Liver X receptor (LXR) belongs to the nuclear receptor family and has two isoforms, LXRα and LXRβ. LXR controls lipid metabolism by regulating the expression of related genes through dimerization with retinoid X receptors (RXRs) (Savla et al., 2022). The expression of LXR varies in different tissues. LXRα is highly expressed in tissues with high lipid metabolism, such as liver, lung, and adipose tissue. LXRβ is widely expressed in distinct tissues. Synthetic LXR agonists have been at the center of active research by pharmaceutical companies, not only because they can combat atherosclerosis, but also due to their potential applications for treatment of other metabolic or neurological disorders as well as cancer (Viennois et al., 2011). Accumulating evidence have shown that the activation of LXR can effectively prevent the development of atherosclerosis by modulating lipid metabolism and inflammation in atherosclerotic plaques (Steffensen et al., 2013; Savla et al., 2022). However, the synthetic ligands that have been developed so far lead to elevated blood triglyceride (TG) levels, fatty liver, and neurological disorders (Schultz et al., 2000; Cave et al., 2016). Of note, targeted drug delivery system may successfully reduce these side effects.

Well-designed nanocarriers can target special tissue and/or cells through specific interaction with biomacromolecules and receptors (Liu et al., 2022; Bhaladhare and Bhattacharjee, 2023). Since the approval of adriamycin liposomes in 1995, nearly 250 kinds of nanotherapeutic drugs have been studied in preclinical or clinical research, especially in cancer therapy (Zhang et al., 2015; Xu et al., 2023). It is worth noting that there are increasing nanotherapeutic studies focusing on atherosclerosis therapy (Soumya and Raghu, 2023). With the development of nanotechnology, nanocarriers can effectively target macrophages in atherosclerosis, enhancing therapeutic effects and reducing the side effects of loaded drugs. Over the last decades, researchers tried to deliver LXR agonists to macrophages within atherosclerotic plaques by distinct nanocarriers, which promote intracellular cholesterol efflux without inducing fatty liver, providing new opportunities for treatment of atherosclerosis by LXR agonists. In this review, we will summarize the advances in the field of macrophage-targeted nanotherapeutic strategies that have been used for therapy of atherosclerosis by focusing on LXR agonists. To make the whole article readable, we will briefly review the roles of macrophages in atherosclerosis and mechanisms of action of LXR agonists. The literatures are searching results of PubMed and Web of Science mainly using “macrophage, atherosclerosis, LXR agonist” as keywords.

## Macrophages in atherosclerosis

**FIGURE 1:**
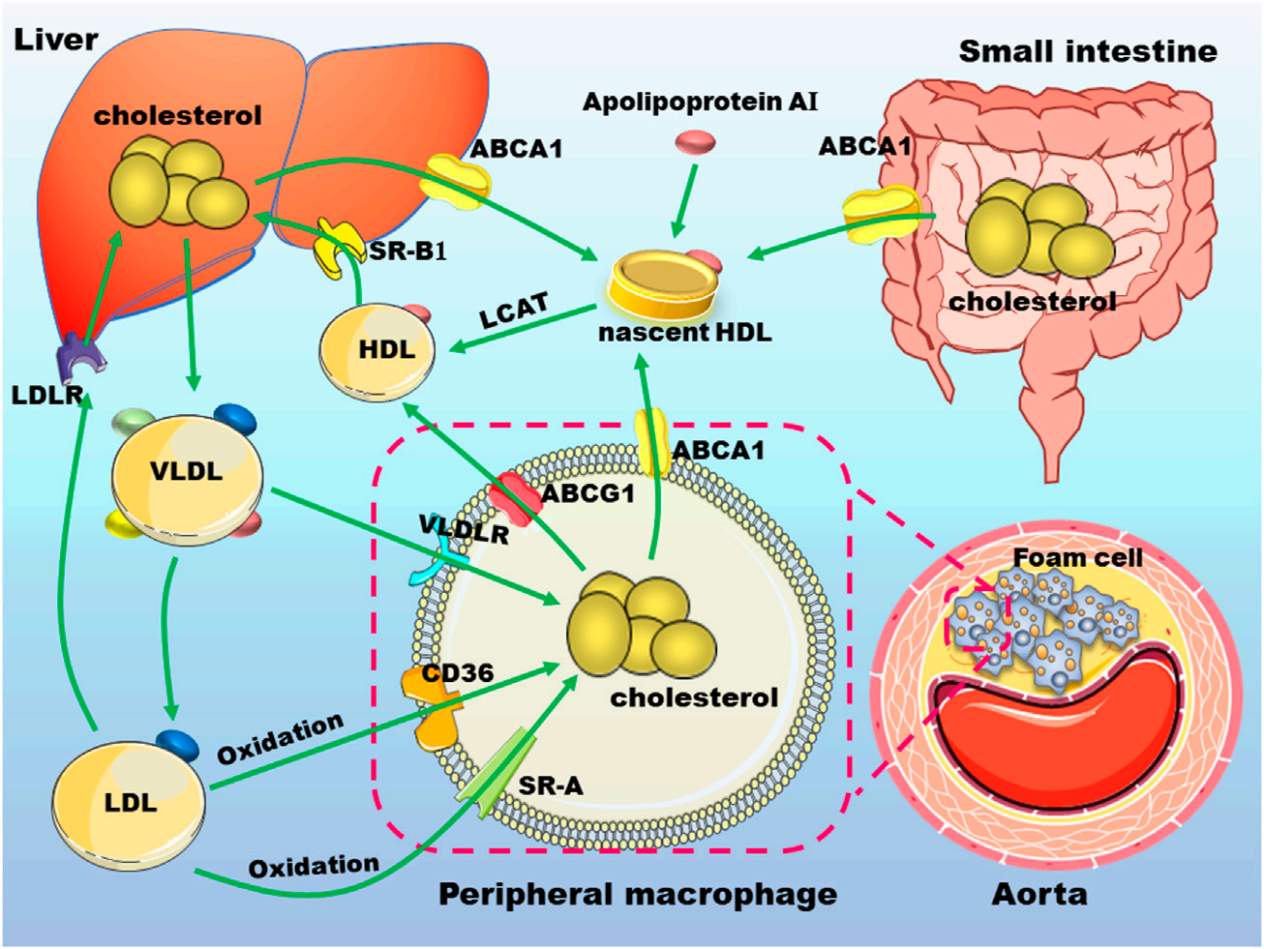
Cholesterol metabolism and macrophages in atherosclerosis. Nascent high-density lipoprotein (HDL) particles derived from apolipoprotein A-I (apo A-I) that is secreted either by liver or small intestine, uptake cholesterol from different cell types, including macrophages *via* ATP binding cassette transporter (ABC) A1. ABCG1 mediates cholesterol transport from peripheral cells to mature HDL. HDL-cholesterol can be esterified by lecithin-cholesterol acyltransferase (LCAT) to promote the formation of mature HDL. HDL is absorbed by hepatocytes mainly by scavenger receptor, class B type 1 and is metabolized in the liver. Hepatic synthesized very low-density lipoprotein (VLDL) particles can be transformed to low-density lipoprotein (LDL). These lipoproteins can be uptake by macrophages through receptors such as VLDL receptor (VLDLR), cluster of differentiation 36 (CD36), scavenger receptor A (SR-A). The excess accumulation of lipids in macrophages under the arterial endothelium will promote the occurrence and development of atherosclerosis. These abbreviations are suitable for the following figures.

**FIGURE 2:**
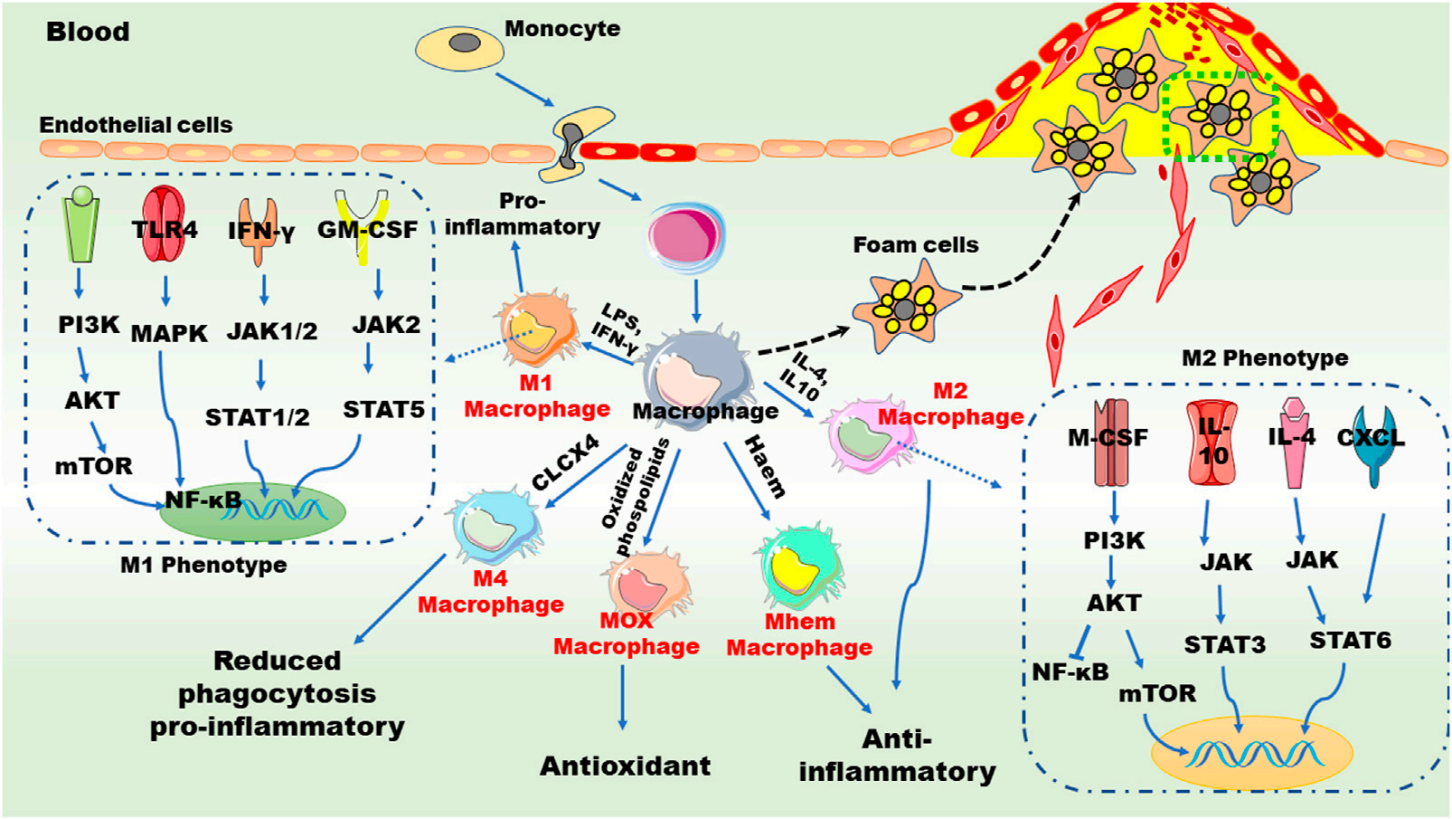
Macrophages and inflammation in atherogenesis. Chronic inflammation promotes the accumulation of monocytes on the surface of endothelial cells of the aorta and induces monocytes transition and transformation. Granulocyte-macrophage colony-stimulating factor and macrophage colony-stimulating factor mediate the polarization of M1 and M2 macrophages, respectively. The polarization of M1 macrophages is promoted by pro-inflammatory factors, such as tumor necrosis factor (TNF), interleukin (IL)-6, monocyte chemoattractant protein 1, and interferon-γ (IFN-γ), while the polarization of M2 macrophages is mediated by anti-inflammatory factors such as IL-4, IL-10, and transforming growth factor *β*. Different signaling pathways are involved in these phenotype shifts. Furthermore, monocyte/macrophage can be differentiated into Mox, M4 and Mhem subtypes upon distinct stimuli. AKT: serine/threonine-protein kinases; CXCL: chemokine (C-X-C motif) ligand; GM-CSF: granulocyte-macrophage colony stimulating factor; JAK: janus kinase; LPS, lipopolysaccharide; MAPK: mitogen activated protein kinases; M-CSF: macrophage colony stimulating factor; mTOR: mechanistic target of rapamycin; NF-κB: nuclear factor kappa-B; PI3K: phosphoinositide 3-kinase; STAT: signal transducers and activators of transcription; TLR: toll-like receptors. These abbreviations are suitable for the following figures.

The pathogenic mechanism of atherosclerosis involved the persistent injuries induced by accumulation of LDL and inflammatory factors resulting in irreparable damage to the structure and function of vascular endothelium. And then, the damaged endothelial cells release monocyte chemokines to recruit large numbers of inflammatory cells which will release inflammatory factors to further accelerate disease progression (Björkegren and Lusis, 2022). Subsequently, LDL in the blood vessels is modified to oxidized LDL (ox-LDL) and can be specifically recognized and taken up by macrophages of monocyte origin to form foam cells ultimately (Lin et al., 2021). In the following sections, we will briefly review the actions of macrophages in lipid metabolism (Figure 1) and inflammation (Figure 2) in atherosclerosis.

### Macrophages and lipid metabolism in atherosclerosis

Foam cells in atherosclerotic plaques are derived from macrophages cells and vascular smooth muscle cells. Macrophages play a central role in the occurrence and development of atherosclerotic CVD (Barrett, 2021; Lin et al., 2021). Cholesterol in normal macrophages come from the internalization of plasma lipoproteins, endocytosis of apoptotic cells, and intracellular cholesterol synthesis. Macrophages absorb cholesterol in lipoproteins, including high-density lipoprotein (HDL) and LDL, through related receptors such as cluster of differentiation 36 (CD36), scavenger receptor, class B type 1 (SR-B1), and LDL receptor (LDLR). As an important scavenger receptor in macrophages, CD36 absorbs ox-LDL and promotes the formation of foam cells (Yang et al., 2018; Vazquez et al., 2020). SR-B1 is a multiligand membrane receptor protein that function as physiologically related HDL receptor (Meslier et al., 2020). The first step in SR-B1-mediated selective uptake of cholesterol ester (CE) is the binding of CE-rich lipoproteins to the extracellular domain of SR-B1. After binding, CE moves down the concentration gradient in HDL particles to cell plasma membranes. When CE is separated from HDL, HDL is released into circulation again to continue peripheral cholesterol transport. Within the cell, CE hydrolase hydrolyzes CE to free cholesterol. Other lipids in HDL particles, such as free cholesterol and TG, are also selectively transferred to macrophages (Shen et al., 2018; Straub, 2019).

Macrophages excrete excess cholesterol to extracellular receptors or convert cholesterol into CE and store it as lipid droplets, thus avoiding the cytotoxic effect of cholesterol accumulation. Several pathways are involved in cholesterol efflux from macrophages: 1) adenosine triphosphate binding cassette A1 (ABCA1) mediated cholesterol efflux to lipid-free apolipoprotein (apo), especially apoA-I; 2) unidirectional cholesterol efflux to mature HDL particles mediated by ABCG1; and 3) passive diffusion of cholesterol promoted by SR-B1 to mature HDL particles (Shen et al., 2018; Matsuo., 2021). ABCA1 and ABCG1 play a vital role in regulating cholesterol homeostasis in macrophages (Skarda et al., 2021). Of note, functions of ABCA1 and ABCG1 need ATP to transport free cholesterol from endoplasmic reticulum to cell membrane. ABCA1-mediates efflux of cholesterol and phosphatidylcholine to lipid-free ApoA-I, promoting the generation of newborn nascent HDL, and ABCG1 mediates the outflow of cholesterol, phosphatidylcholine, and sphingomyelin to nascent HDL and mature HDL. Dysfunction of ABCA1 and ABCG1 significantly reduces serum HDL levels, thereby greatly impairing cholesterol and lipid transport (Matsuo., 2021; Wu et al., 2022). Of note, SR-B1 not only mediates the selective entry of HDL into macrophages but also promotes the outflow of intracellular free cholesterol, preventing free cholesterol from accumulation in the arterial wall (Figure 1). Therefore, macrophages play a pivotal role in reverse cholesterol transport (RCT). Abnormal RCT accelerates the occurrence and development of atherosclerosis (Rohatgi, 2018; Ma et al., 2021).

### Macrophages and inflammation in atherosclerosis

Macrophages contribute to the sustained local inflammatory response by secreting chemokines/cytokines as well as factors leading to oxidative stress (Khoury et al., 2021). A growing body of evidence suggests that resident macrophages can proliferate under microenvironment stimuli, such as oxidized lipids accumulation, inflammation response, and cytokine secretion which will in turn influence plaque microenvironment (Lin et al., 2021; Wang et al., 2023). For instance, in the atherosclerotic plaque environment, macrophages can also be initiated by oxidized lipids, induce a “foamy” state of activation, and exhibit an anti-inflammatory phenotype (Kuznetsova et al., 2020). Interestingly, macrophages can differentiate into distinct subtypes with different functions under different stimuli (Khoury et al., 2021). According to the dichotomy, macrophages are divided into two classes: classically activated pro-inflammatory M1 macrophages and alternatively activated anti-inflammatory M2 macrophages (Naumann et al., 2023). As shown in Figure 2, pro-inflammatory M1 macrophages are typically triggered by T helper (Th) 1 cytokines and lead to an inflammatory response, whereas M2 macrophages are mainly induced by Th2 cytokines and alleviate inflammation *via* producing anti-inflammatory cytokines (Liu et al., 2021).

Of note, high levels of the glycoprotein Ly6C monocytes in mice, known as CD14 (CD14^++^/CD16_−_ subtype) in humans, differentiate into M1 macrophages, while low levels of the glycoprotein Ly6C monocytes differentiate into M2 macrophages (Liu et al., 2021; Yang et al., 2022). In the arterial intima, monocytes differentiate into macrophages by multiple pro-differentiation factors (Lin et al., 2021). Granulocyte-macrophage colony-stimulating factor and macrophage colony-stimulating factor mediate the polarization of M1 and M2 macrophages, respectively (Jiemy et al., 2020; Lin et al., 2021). Macrophages engulf modified LDL in the intima to form lipid-laden foam cells, giving rise to early atherosclerotic lesions. Generally, macrophages exert their atheroprotective effects through endocytic clearance of lipoprotein deposits and efferocytosis (phagocytosis clearance of apoptotic cells), thereby counteracting inflammatory processes involved in plaque formation (Yin et al., 2020; Kong et al., 2022). If the pro-inflammatory state persists, the atherosclerotic lesions progress to an advanced stage characterized by increased macrophage apoptosis and defective clearance of apoptotic cells (Lin et al., 2021; Chen et al., 2022).

Present data suggest that the ratio of pro-inflammatory M1 to anti-inflammatory M2 macrophages, within the atherosclerosis plaques may determine the progression and regression of atherosclerosis (Lin et al., 2021). M1 macrophages are the major inflammatory macrophage cell population in lipid cores of atherosclerotic plaques (Zang et al., 2021). M2 macrophages can be divided into three subtypes, namely, M2a, M2b and M2c. The polarization of M1 macrophages is promoted by pro-inflammatory factors, such as tumor necrosis factor (TNF), interleukin (IL)-6, monocyte chemoattractant protein 1, and interferon-γ, while the polarization of M2 macrophages is mediated by anti-inflammatory factors such as IL-4, IL-10, and transforming growth factor *β* (Lin et al., 2021; Yang et al., 2022). Interestingly, TNF-α decreases the expression of ABCA1, exaggerating foam cell formation (Hu et al., 2021). IL-10 secreted by M2 type macrophages not only stimulates the function of ABCA1 and ABCG1 but also inhibits CD36-mediated ox-LDL uptake by macrophages (Hu et al., 2021; Li H et al., 2022). Two main signaling pathways, the Akt/mTORC/LXR pathway and the JAK/STAT6 pathway, are two major signaling pathway for M2 polarization. (Bi et al., 2019; Fang et al., 2022). Besides, Th2 cell-secreted molecules, such as IL-4, can also induce the differentiation of macrophages toward M2 phenotype, thereby alleviating inflammation by inhibiting the mitogen-activated protein kinase signaling pathway (Zhao et al., 2016). The shift of macrophages from M1 to M2 phenotype makes it possible to maintain a basal anti-inflammatory environment in atherosclerosis plaques.

Furthermore, additional plaque-specific macrophage phenotypes have been recently identified (Figure 2), including oxidized phospholipid-induced macrophages (Mox), chemokine (C-X-C motif) ligand 4 (CXC-4) or platelet factor 4-induced macrophages (M4), erythrocyte and hemoglobin-induced macrophages (HA-mac, M(Hb), and Mhem) (Maretti-Mira et al., 2021; Nakai, 2021). Mox macrophages, a pro-atherogenic subset induced by oxidized phospholipids, express redox-regulatory genes as well as pro-inflammatory genes *via* Toll-like receptor 2 and display a proatherogenic ability (Li Z et al., 2022). In atherosclerotic lesions of LDLR-deficient (LDLR^*−/−*^) mice, Mox macrophages are extensively distributed in plaque and account for 30% of the total macrophages (Li H et al., 2022). Notably, the phagocytosis and migration capacities of Mox macrophages are inferior to M1 and M2 subsets. M4 macrophages are polarized by CXC-4 (Domschke and Gleissner, 2019) and they are associated with reduced phagocytosis and elevated production of inflammatory cytokines and molecules, such as IL-6, TNF-α, and MMP-7 (Skuratovskaia et al., 2020). The M4 subset is dominatingly presented in the adventitia and intima of human arteries to trigger inflammation and promote plaque instability (Lin et al., 2021). Hemorrhage-residing Mhem macrophages participate in hemo-globin clearance *via* phagocytosis of erythrocyte and improve cholesterol efflux by enhancing the expression of cholesterol transporters, ABCA1 and ABCG1, through LXRα and LXRβ (Li Z et al., 2022; Lin et al., 2021; Kong et al., 2022).

## LXR agonists for therapy of atherosclerosis

Studies have shown that when mice are fed a high-cholesterol diet, the deficiency of LXR leads to pathological accumulation of cholesterol in the liver. When mice are fed a normal chow diet, the loss of liver LXR mainly leads to metabolic disorder of fatty acids and phospholipids (Bideyan et al., 2022). LXR are involved in reduction of intestinal cholesterol absorption and increase of intestinal excretion *via* down-regulating Niemann-Pick C1-like 1 and elevating ABCG5/8 (Muscoli et al., 2022). Furthermore, LXR participate in the repair and anti-inflammation of vascular cells through multiple pathways (Xu et al., 2020). However, LXR agonists lead to elevated blood TG levels, resulting in significant fat accumulation in the liver, leading to hepatic steatosis and even liver lesions, which limit their application (Schultz et al., 2000).

### Mechanisms of action of LXR signaling pathway

LXR can be activated with oxysterols, which are derivatives of cholesterol. Upon activation, LXRα and LXRβ interact with RXRs to form specific heterodimers, which bind to promoters of multiple downstream genes involved in lipid metabolism (Abe et al., 2022). For instance, LXR activation promote the expression of ABC transporters, which play important roles in macrophage cholesterol efflux, hepatic and intestinal lipid homeostasis (Tanigawa et al., 2009; Van der Velde, 2010; Peng et al., 2019). As discussed above, activation of ABCA1 and ABCG1 through LXR enhances macrophage cholesterol efflux and promotes the generation of mature HDL and upregulation of RCT (Kasbi Chadli et al., 2013; Guo Y et al., 2018). The expression of ABCA1 and ABCG1 is mainly regulated by LXRα. Several lines of evidence have shown that ABCG1 upregulation promotes cholesterol transport from peripheral cells to HDL and inhibits transformation of macrophages to foam cells (Matsuo., 2021; Zhang X et al., 2021). Moreover, LXR upregulate the specific expression of ABCG5 and ABCG8 on the surface of hepatocytes and intestinal cells. ABCG5 and ABCG8 form a heterodimer that affects the hepatic cholesterol excretion and intestinal cholesterol absorption. LXR also upregulate the expression of ApoE, which is involved in transformation and metabolism of lipoproteins and is an important regulator of cholesterol homeostasis (Li et al., 2019). In addition, the expression of NPC1L1 can be downregulated by LXR activation, thus reducing absorption of intestinal cholesterol (Li et al., 2017).

**FIGURE 3:**
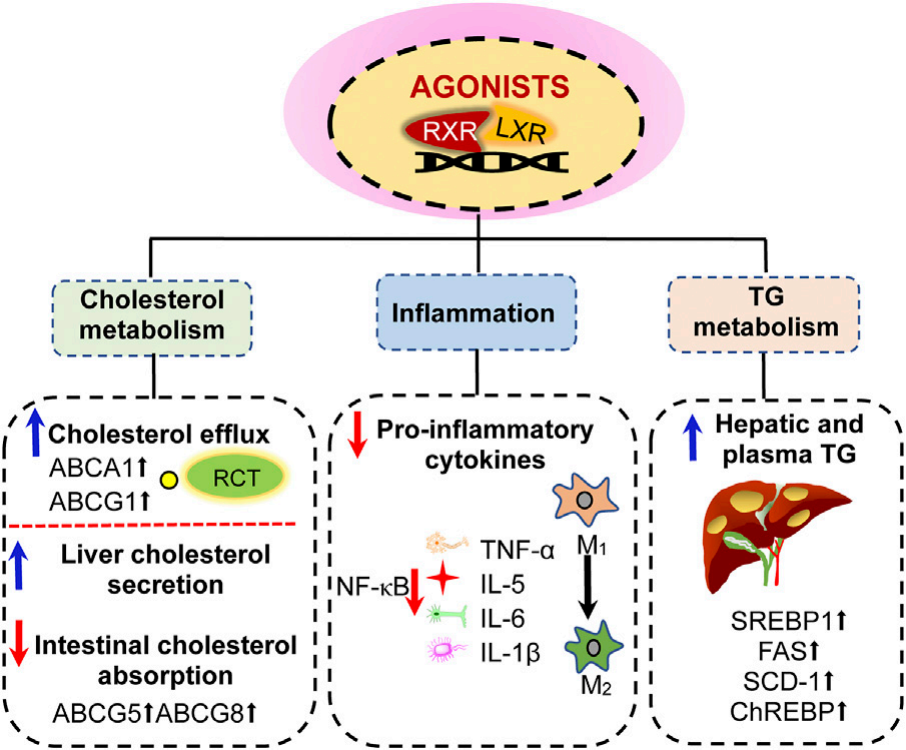
Effects of liver X receptor (LXR) activation. (1) activation of LXR can promote reverse cholesterol transport by upregulating the expression of ABCA1 and ABCG1 genes in peripheral cells, thereby reducing intracellular cholesterol content; LXR activation also promote cholesterol secretion from the liver and small intestine by upregulating the expression of ABCG5 and ABCG8. (2) LXR activation inhibits NF-κB signaling pathway to suppress inflammatory factors, including the expression of TNF-α, IL-5, IL-6, and IL-1β. (3) LXR activation induces hypertriglyceridemia and liver steatosis by enhancing the expression of sterol regulatory element-binding protein-1c (SREBP-1c), fatty acid synthase (FAS), stearoyl-coenzyme A dehydrogenase-1 (SCD-1), and carbohydrate response element-binding protein (ChREBP). RXR, retinoid X receptors; TG, triglyceride.

It is worth noting that LXR not only play a key role in cholesterol metabolism but also elevate hepatic lipogenesis (Figure 3). Upon activation, LXR induce the expression of adipogenesis-related genes, such as sterol regulatory element-binding protein-1c (SREBP-1c), fatty acid synthase, stearoyl-coenzyme A dehydrogenase-1 and carbohydrate response element-binding protein, resulting in elevated plasma and hepatic TG levels (Schultz et al., 2000). SREBP-1c, the main transcription regulator of fatty acid and TG synthesis, is mainly regulated by LXRα is in liver. As previously reviewed, LXRβ knockout has no effect on the expression of SREBP-1c (Golub et al., 2023). As a cholesterol sensor, LXRα activates SREBP1c to enhance synthesis of fatty acids which are substrates for CE synthesis, thereby alleviating intracellular cholesterol accumulation (WHO, 2023). However, this process induces the main side effects of LXR activation.

In addition, LXR activation influences the biological properties of related inflammatory cells and inhibits gene expression of inflammatory mediators (Figure 3). According to the pathological formation process of atherosclerosis, the inflammatory cells mainly include vessel endothelial cells, macrophages, T lymphocytes, and smooth muscle cells (Ross, 1999). LXR are expressed in these cells and their activation can modulate the inflammatory states. For instance, the endogenous LXR activator, desmosterol, suppresses inflammatory genes in foam cells of atherosclerotic plaques (Spann et al., 2012; Zhang S et al., 2021). It has been demonstrated that LXR activation inhibits the nuclear factor kappa B signaling pathway (Marathe et al., 2006). Furthermore, LXR activation inhibits the production of a series of pro-inflammatory factors which are stimulated by bacteria, bacterial endotoxin lipopolysaccharide (LPS), TNF-α, and IL-1β. LXR agonists play an important regulatory role in inhibiting the expression of proinflammatory genes in macrophages and other types of cells (Findeisen et al., 2022). LXRα can selectively regulate the expression of genes related to apoptosis and leukocyte migration. Furthermore, LXRα plays an important role in the development of autoimmune hepatitis (Khoury et al., 2020). The function of LXRβ is closely related to lymphocyte differentiation and selection.

The coupling process between the agonist-bound LXR and the small ubiquitin-related modifier (SUMO) is called SUMOylation. In macrophages, SUMOylated LXR interacts with actin-binding protein CORONIN 2A to prevent actin from recruiting inflammatory gene promoters (Huang et al., 2011). Upon interferon-γ stimuli, both SUMOylated LXRα and LXRβ can inhibit the transcriptional response of mouse macrophages (Glaría et al., 2020). Of note, LXR activation inhibits the production of pro-inflammatory cytokine IL-18 directly and indirectly. LXR indirectly induces the expression of IL-18 binding protein, which is an effective endogenous inhibitor of IL-18 in mice and human systems, by up-regulating interferon regulatory factor 8 (Pourcet et al., 2016). Moreover, LXR agonists promote the expansion of regulatory T cells and inhibit the differentiation of Th17 cells that can secrete pro-inflammatory factor IL-17 in mice and humans (Yasuda et al., 2019).

### Anti-atherosclerotic effects of LXR agonists

**FIGURE 4:**
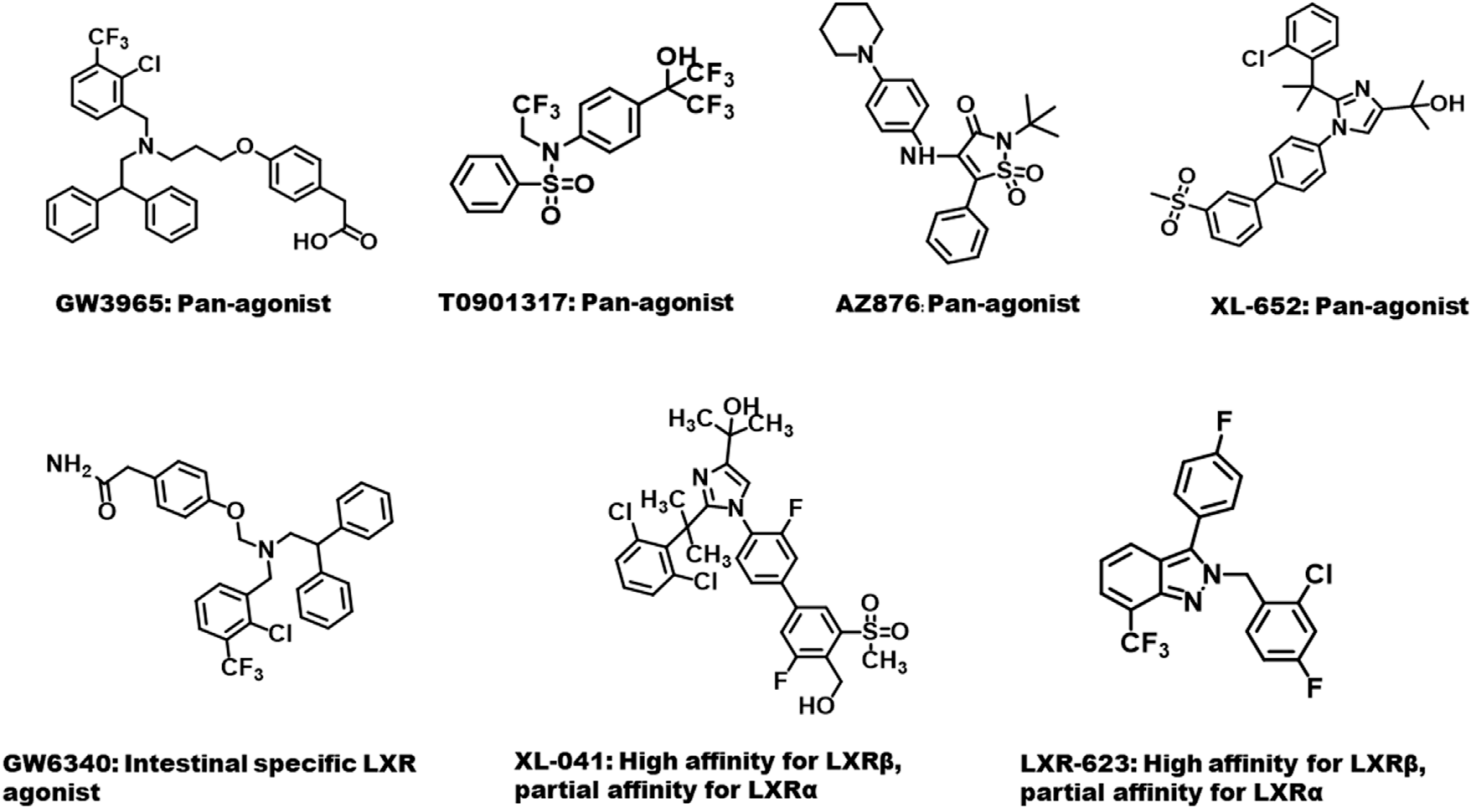
Structures of several mostly studied synthetic liver X receptor agonists.

Given the pivotal roles of LXR in inhibiting inflammation and promoting RCT, LXR agonists can effectively prevent the development of atherosclerosis. As shown in Figure 4, some synthetic LXR agonists have been reported. The synthetic LXR agonist GW3965 upregulates the expression of ABCA1 and ABCG1, thereby promoting cholesterol efflux from murine RAW264.7 macrophages and reducing aortic plaque formation in LDLR^−/−^ mice (Venkateswaran et al., 2000; Joseph et al., 2002). The anti-atherosclerosis effects of the synthetic LXR agonists GW3965 and T0901317 have been further demonstrated in other atherosclerosis models, such as apoE-deficient (apoE^*−/−*^) and LDLR^*−/−*^ mice (Joseph et al., 2002; Terasaka et al., 2003). In 2006, another study demonstrates that GW3965 promotes cholesterol efflux from mouse macrophages and enhances cholesterol secretion from liver, inhibiting atherosclerosis by improving RCT (Wang et al., 2007). As shown in Table 1, LXR agonists have demonstrated powerful anti-atherosclerosis effects in distinct animal models (Rasheed and Cummins, 2018).

**TABLE 1:** Summary of synthetic LXR agonists for therapy of atherosclerosis.

LXR agonists	Model and dosage	Beneficial effects	Disadvantages	References
GW3965	LDLR^−/−^ mice, 1 mg/kg and 10 mg/kg ApoE^−/−^ mice, 10 mg/kg; Feed for 12 weeks	Reduction in aortic root injury; In ApoE^−/−^ mice, TC levels and HDL cholesterol levels were unchanged	Elevated plasma TG significantly; Tolerance or compensation may occur with chronic GW3965 administration	Joseph et al. (2002)
	LDLR^−/−^ mice treated with GW3965(1μ mol/L), once every 2 weeks for 8 weeks	Decrease the endothelial defects associated	Upregulation of hypertriglyceridemia	Rasheed et al. (2021)
T0901317	LDLR^−/−^ mic, 0.2, 0.5, 1, 2 mg/kg T0901317; Gavage; Once a day for 2 weeks	Produced a 54%, 75% and 94% decline in atherosclerosis plaque area; Inhibit expression of pro-inflammatory molecules perhaps by limiting the activation of NF-κB	Elevated VLDL-CH and total TG significantly	Schultz et al., (2000), Peng et al., (2009)
	ApoE^−/−^ mice, T090317(0.5 mg/kg) in combination with notch receptor inhibitors (1.0 mg/kg), feed for 4 weeks	Reduced liver cholesterol and TG, upregulation of ABCA and RCT.	Elevated serum alanine amino-transferase aspartate aminotransferase and hepatic injury slightly	Hao et al. (2020)
LXR-623	LDLR^−/−^ mice, 15 mg/kg/day or 50 mg/kg/day; Golden Gopher, 20, 60, 120 mg/kg/day; Crab-eating macaques, 1.15, 2.50 and 50 mg/kg/day	Reduced area of aortic arch injury, plasma total cholesterol and LDLC; not elevated plasma and liver cholesterol and TG; less potent for inducing SREBP1c and associated TG accumulation in liver cells	It was discontinued due to significant loss of certain neurons in the brain in the central nervous system	Katz et al., (2009), Quinet et al., (2009), Wang et al., (2017)
	C57BL/6 mice, tunicamycin 1 mg/kg and LXR-623 15 mg/kg, injected intraperitoneally, observed after 48 h	Elevated ABCA1 and ABCG1 in hepatocytes and macrophages.	Elevated liver total cholesterol and TG slightly	Wang T et al. (2020)
GW6340	Wild-type mice, 30 mg/kg; primary macrophages deficient in LXRα/β	Upregulation of ABCA1, ABCG5 and ABCG8. It does not modulate TG content	Elevated total TG.	Yasuda et al. (2010)
XL-652	Male cynomolgus	Upregulation of ABCA1 and ABCG1	Elevated plasma TG and LDL cholesterol cannot be completely avoided	Kirchgessner et al. (2015)
	Monkeys. 1 mg/kg; or 0.3, 1, and 10 mg/kg/day			
XL-041	C57BL/6J mice, 0.03, 0.1, 1, or 3 mg/kg/day; LDLR^−/−^mice, 0.03–3 mg/day; Male cynomolgus Monkeys, 0.1, 0.3, 1, or 3 mg/kg/day	Upregulation of RCT by 70%	Elevated plasma TG. Terminated due to undisclosed reasons in 2013	Kirchgessner et al. (2016)
CS8080	Healthy Volunteers, 1, 3, 10, 20, 50 or 100 mg/day	No study results posted on llinicalTrials.gov for this study	By late 2008, the project was terminated due to undisclosed safety concerns	NCT00796575. 2008. Available from: clinicaltrials.gov
AZ876	Female ApoE*3 leiden transgenic mice. 5 mmol/kg/day and 20 mmol/kg/day	AZ876 20 mmol/kg increased total lipids by 75%, reduced lesion area by 91%, and had anti-inflammatory effects; 5 mmol/kg/day decreased lesion area by 47%	Elevated liver TG level	van der Hoorn et al. (2011)
IMB-808	RAW264.7 macrophages, HepG2, HEK293T, Caco-2 cells and THP-1 cells. From 0.001 μM to 30 μM	Upregulation of ABCA1, ABCG1, apoE, ABCG8 and ABCG5. Downregulation of NPC1L1. Promotes cholesterol efflux, reduces cellular lipid accumulation and almost does not induce lipogenic gene expression	Not reported	Li et al. (2017)

Abbreviations: ABC, ATP-binding cassette transporter; ApoE, apolipoprotein E; ApoE^−/−^, Apolipoprotein E-deficient; HDL, High-density lipoprotein; LDL, Low-density lipoprotein; LDLR^−/−^, Low-density lipoprotein receptor-deficient; LXR, Liver X receptor; NF-κB, Nuclear factor kappa-B; NPC1L1, Niemann-Pick C1 like 1; RCT, Reverse cholesterol transport; SREBP-1c, Sterol regulatory element-binding protein-1c; TG, Triglyceride; VLDL-C, Very low-density lipoprotein cholesterol.

However, LXR pan-agonists are found to activate fatty genes in liver, such as fatty acid synthase and acetyl-Co A carboxylase 1, which induce hypertriglyceridemia and liver steatosis (Schultz et al., 2000). Furthermore, LXR agonists downregulate the expression of apoA5, resulting in hypertriglyceridemia by modulating ANGPTL3/8-mediated LPL activity (Chen et al., 2021). It seems that selective activation of LXRβ or tissue-selective activation are more feasible for treatment of atherosclerosis. For instance, the intestinal LXR agonist GW6340 activates the expression of intestinal ABCA1, ABCG5, and ABCG8 with little effect on hepatic LXR target genes (Yasuda et al., 2010). The selective LXRβ agonist LXR-623 (WAY 252623) improves RCT without boosting fatty acid synthesis in the liver. However, the phase I clinical trial of LXR-623 has been terminated due to its adverse reactions in the central nervous system (Katz et al., 2009). Moreover, several other compounds, including CS8080, BMS-779788 (XL-652), and BMS-852927 (XL-041), were terminated due to their unexpected side effects after entering phase I clinical trial (Loren et al., 2013). The side effects of these synthetic LXR agonists are also listed in Table 1. Interestingly, LXR agonists in combination with other drugs may potentially alleviate the side effects of these agonists. For instance, LXR-623 combined with simvastatin induces atherosclerotic plaque regression in New Zealand white rabbits at a lower dose and effectively reduces side effects (Giannarelli et al., 2012). The combination of U0126, a MEK1/2 inhibitor, and LXRα T0901317 synergistically reduces the formation of atherosclerotic plaques and avoids lipogenesis induced by T0901317 (Hayashi et al., 2014). Metformin, a first-line agent for the treatment of type II diabetes, can also synergistically inhibit atherosclerosis and reduce lipogenesis induced by T0901317 (Kim et al., 2015). Although combination therapy may partially reduce the side effects of LXR agonists, the long-term beneficial effects need to be investigated in future and in other animal models.

In addition to lipid-lowering, LXR activation attenuates the expression of inflammatory genes in macrophages (Pourcet et al., 2016). For instance, LXR activation suppresses the expression of inflammatory genes by multiple mechanisms including cis-repression (Thomas et al., 2018), trans-repression (Ghisletti et al., 2007), and cholesterol efflux-mediated regulation (Yvan-Charvet et al., 2008; Ito et al., 2015). Similarly, LXR activation represses inflammatory genes and upregulates cholesterol efflux genes in myelin damage-associated microglia (Berghoff et al., 2021). In addition, macrophages have a favorable homeostatic role in atherosclerosis, helping to clear lipid debris derived from circulating lipoproteins and necrotic cells while tamping down inflammation (Endo-Umeda et al., 2022).

## Targeting macrophages in atherosclerosis using nanocarriers

### Macrophage receptors

**FIGURE 5:**
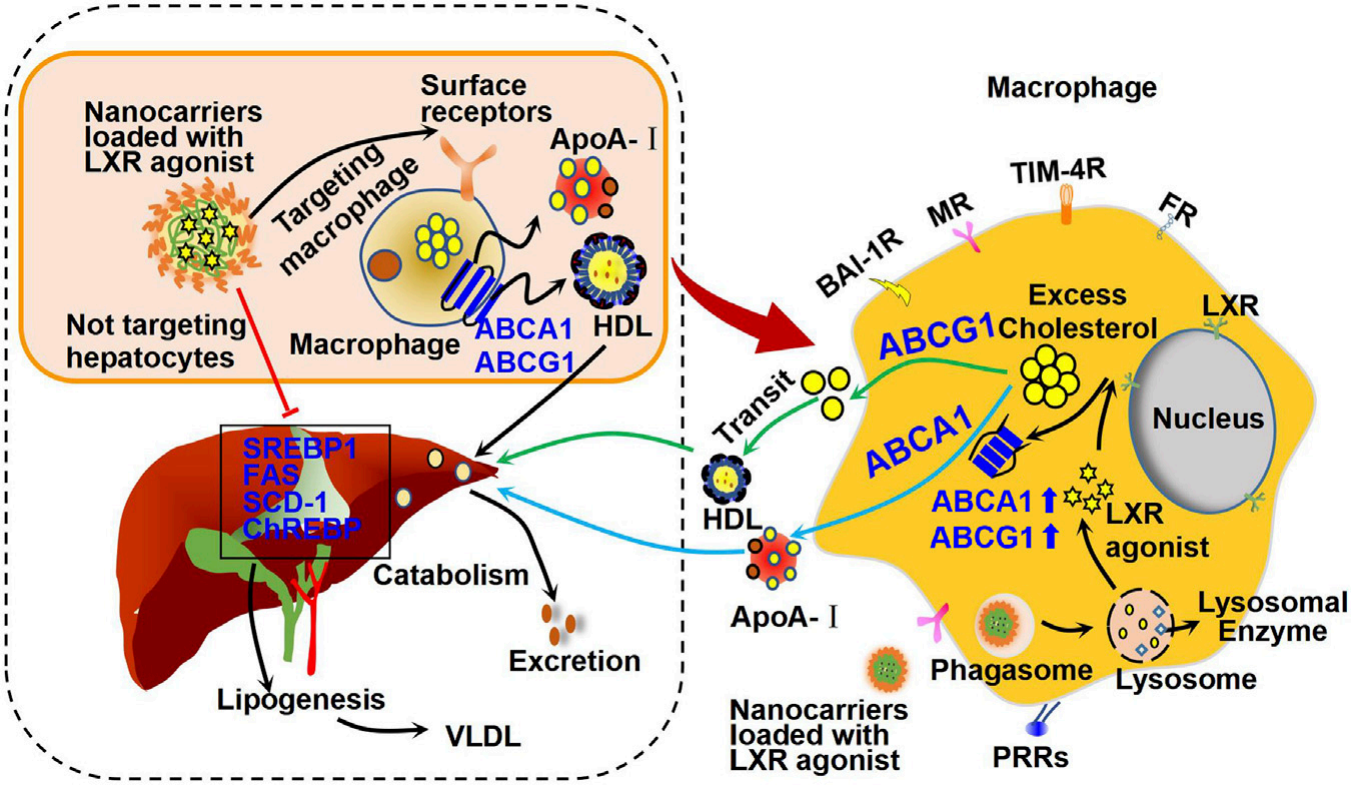
Mechanism of action of macrophage-targeted nanocarriers loaded with LXR agonists. Specially designed nanocarriers loaded with LXR agonists can be recognized and ingested by macrophages through the corresponding surface receptors, such as brain-specific angiogenesis inhibitor 1 receptor (BAI-1R), mannose receptor (MR), T-cell immunoglobulin and mucin domain containing protein 4 receptor (TIM-4R), folate receptor (FR), and pattern recognition receptors (PRRs). Therefore, these nanocarriers may activate cholesterol efflux and reduce inflammation as shown in Figure 3. Importantly, these nanocarriers are supposed to be seldomly absorbed by hepatocytes, and thus they may have minor effect on hepatic lipogenesis genes, including SREBP1, FAS, and SCD-1 as shown in Figure 3. Therefore, these nanocarriers show minor effect on fat accumulation in liver and may reduce side effects of LXR agonists in other organs.

Macrophages play an important role in many biological processes, such as phagocytosis and killing of microorganisms (Fogelman et al., 1988). These biological activities are determined by their surface membrane receptors, including mannose receptor (MR) (Martinez-Pomares, 2012), folate receptor (FR) (Shen et al., 2014), T-cell immunoglobulin and mucin domain containing protein 4 (TIM-4) (Miyanishi et al., 2007), brain-specific angiogenesis inhibitor 1 (BAI-1) (Park et al., 2007), and pattern recognition receptors (PRRs) (Terpstra et al., 1997). These surface receptors of macrophage mediate internalization of exogenous nanoparticles and can be used for designing specific macrophage-targeted drug delivery systems (Figure 5).

MR, an endocytic protein, is a carbohydrate binding receptor that is highly expressed on macrophages (Ezekowitzet et al., 1991). The uptake of mannose glycopolymer was enhanced in M2-polarized macrophages. This carbohydrate-specific receptor has been used in gene vaccine by targeting human dendritic cells and macrophages through the phagocytic pathway (Wattendorf et al., 2008). FR beta (FR-β) is specifically expressed by activated macrophages and has a high affinity for folic acid (Puig-Kroger et al., 2009; Van Der Heijidan et al., 2009). Therefore, a folate-based nanocarrier may deliver therapeutic agents to activated macrophages without affecting normal cells and tissues. BAI-1, a phagocytic receptor, is widely distributed on the surface of macrophages and has an important role in macrophage phagocytosis. Studies have shown that BAI-1 mediates macrophage phagocytosis and can recognize LPS on the surface of microorganisms (Park et al., 2007; Das, et al., 2011). TIM-4, one of the cellular immunoglobulin mucin molecules, is selectively expressed on the surface of macrophages (Kobayashi et al., 2007; Rodriguez-Manzanet et al., 2008; Wong et al., 2010). TIM-4 is also a phosphatidylserine receptor and acts as a receptor involved in macrophage phagocytosis of apoptotic cells (Nurtanio and Yang, 2011). In addition, the PRRs of macrophages recognize modified LDL as damage associated molecular patterns or an ‘eat-me’ signal (Terpstra et al., 1997; Terpstra et al., 1998). Dectin-1, a member of the PRRs family, is expressed in macrophages and is essential for macrophage phagocytosis of yeast by recognizing the cellular component *β*-glucan of yeast (Herre et al., 2004). siRNAs with *β*-glucose shells targeting the dectin-1 receptor of macrophages have been successfully designed for oral treatment of systemic inflammation (Puig-Kröger et al., 2009). In a recent study, liposomes were functionalized with CD47-derived auto peptides and galactose in combination with carriers of “eat me/not eat me” signals to precisely deliver nanomedicines to M2 macrophages (Tang et al., 2021).

Last but not the least, most reported scavenger receptors are highly expressed by phagocytes including macrophages. These receptors can bind ligands of both pathogen- and self-origin and recognize modified LDL particles (such as oxidation or acetylation) (Graversen et al., 2012; Wilkinson and EI Khoury, 2012). In this aspect, Wang et al. reported that multi-walled carbon nanotubes (MWCNTs) cause apoptosis and inflammatory response in a dose-and time-dependent manner (Elgrabli et al., 2008). Apoptosis percentage among MWCNTs-treated RAW264.7 cells are significantly reduced when non-specific inhibitor of scavenger receptor (poly I) and the scavenger receptor-A specific inhibitor (2F8) is employed (Wang et al., 2012). These findings suggest that scavenger receptors can also be explored as a target of macrophages.

### Macrophage-targeted nanocarriers for therapy of atherosclerosis

The rapid development of drug delivery system has provided new hope for alleviating drug side effects and improving the beneficial effects of LXR agonists (Figure 5). After entering the circulation, specially designed nanocarriers loaded with LXR agonists can be recognized and taken up by receptors on the surface of macrophages as mentioned earlier (Chen et al., 2022), but not by hepatocytes, thereby effectively blocking the effect of LXR agonists on hepatic adipogenesis. Upon uptake by macrophages, some macrophages infiltrate into plaques, where they are degraded by lysosomes, releasing LXR agonists that inhibit macrophage-derived foam cell formation and plaque inflammation (Ma et al., 2021). In the following, we will focus on the advances in macrophage-targeted nanocarriers that are loaded with LXR agonists for therapy of atherosclerosis (Table 2).

**TABLE 2:** Macrophage-targeted nanocarriers loaded with synthetic LXR agonists for therapy of atherosclerosis.

Nanocarriers	Target	Model and dosage	Advantages	Disadvantages	References
PLA-DSPE-mPEG1000/DLPC/Col IV-DSPE-PEG2000-GW3965-NPs	Collagen type IV of atherosclerotic plaque	LDLR^*−/−*^ mice, 8 mg/kg GW3965, intravenous, twice weekly for 5 weeks	Macrophage content reduced by ∼30%; curative effect was enhanced by ∼18% without inducing serum TG compared to GW3965 alone; Excellent physical stability and biocompatibility	Minor hepatotoxicity; Cumbersome preparation steps	Yu et al. (2017)
PLGA-b-PEG-GW3965-NPs	Enhanced permeability and retention produced by the “leaky” endothelium	LDLR^*−/−*^ mice, 10 mg/kg GW3965, retro-orbital injection, three times weekly for 2 weeks	The atherosclerotic area reduced by 50%; reduced hepatic TC and TG levels; Reduced inflammation; High biodegradability; Excellent biocompatibility	Entrapment and loading efficacy are not very high of nanocarriers	Zhang et al. (2015)
PS-PLGA-b-PEG-GW3965-NPs	TIM-4 receptor of macrophage	LDLR^*−/−*^ mice, 10 mg/kg of GW3965, retro-orbital injection, three times weekly for 2 weeks	The atherosclerotic area reduced by 40%; reduced hepatic TG levels; Reduced inflammation; High biodegradability; Excellent biocompatibility	Raised hepatic TC levels; Entrapment and loading efficacy are not very high	Zhang et al. (2015)
PLGA-HDL-NPs	Natural targeting by mimicking the HDL nanoparticles	ApoE^*−/−*^ mice, 0.2 mL of PLGA-HDL (10 mg/mL)	Improved cellular cholesterol efflux to PLGA–HDL; Controlled release properties	Did not show specific targeting for macrophages; Complex design and cumbersome steps	Sanchez-Gaytan et al. (2015)
mDNP-T0901317-NPs	Mannose receptor of macrophage	LDLR^*−/−*^ mice, 200 μg per mouse. Tail vein injection, weekly for 4 weeks	Reduced atherosclerotic plaque progression, plaque necrosis, and plaque inflammation without affecting hepatic lipogenic genes or plasma lipids; High buffering capacity, extend the circulation time in the blood	Reducing atherosclerotic plaque that has already formed does not prevent plaque formation at all; Cumbersome preparation steps	He et al. (2018)
Lyp-1-GW3965-liposomes	P32 receptor of foam cells	LDLR^*−/−*^ mice, 6.5 mg/mL GW3965, intravenous injections, twice a week for 5 weeks	The macrophage content reduced two-fold; stabilized atherosclerotic plaques without increasing plasma or hepatic lipid levels; Significant ability to penetrate atherosclerotic plaques	Uptake of liposomes by Kupffer cells in the liver; P32 target has been found to be expressed not only in macrophages	Benne et al. (2020)
D-napg-gffy-T0901317- nanohydrogel	Scavenger receptor class A of macrophage	C57BL/6J mice and ApoE^*−/−*^ mice, 5 mg/kg and 15 mg/kg, subcutaneous injection, once every 3 days for 16 days	Reversed advanced lesions without inducing hypertriglyceridaemia, fatty liver and other liver injuries; inhibited formation of foam cells and production of pro-inflammatory cytokines; Nanocarriers have high stability *in vivo*	The curative effect on atherosclerosis was similar as oral T0901317; Small amounts of nanocarriers can be degraded by extracellular proteinase K	Ma et al. (2021)
SHDL-T0901317-NPs	Cholesterol receptor in atherosclerotic plaques	ApoE^*−/−*^ mice, 1.5 mg/kg, intraperitoneal injection, three times weekly	Significantly reduced atherogenesis and hypertriglyceridemia compared to T0901317 alone; Small size, high purity of nanoparticles	Minor hepatotoxicity	Yuan et al. (2021)
ApoA-I(22A)-sHDL-T0901317-NPs	Cholesterol receptors in atherosclerotic plaques	C57BL/6J mice and ApoE^*−/−*^ mice, 1.5 mg/kg T0901317, intraperitoneal injection, three times weekly for 6 weeks	Upregulation of ABCA1 and ABCG1. Had no influence on hepatic or serum TG levels; Good tolerability; long mean half-life	Difficult and expensive for preparation	Guo Y et al. (2018)
ApoA-Ⅰ protein-Phospholipids-GW3965-NPs	Cholesterol receptors of macrophages in atherosclerosis	ApoE^*−/−*^ mice. Loaded with GW3965 (10 mg/kg). Injection every 2 days for 8 days	Small size (30 nm), accumulated in aortic plaques and exhibited local anti-inflammatory effects. Abolished liver toxicity; Long blood half-life	Did not show advantage compared to free drug in treatment of atherosclerosis; Cumbersome preparation steps	Tang et al. (2016)

Abbreviations: ABC, ATP-binding cassette transporter; ApoA-Ⅰ, apolipoprotein A-Ⅰ; ApoA-I(22A)-sHDL, a synthetic apoA-I mimetic peptide (22-amino acid peptide, 22A) is utilized to formulate sHDL; ApoE^*−/−*^, Apolipoprotein E-deficient; D-napg-gffy, naphthylacetic acid modified D-enantiomeric-glycine-phenylalanine-phenylalanine-tyrosine; LDLR^*−/−*^, Low-density lipoprotein receptor-deficient; Lyp-1, the cyclic peptide Lyp-1 (CGNKRTRGC); mDNP, mannose functionalized dendrimer nanoparticles; NPs, nanoparticles; PLA-DSPE-mPEG1000/DLPC/Col IV-DSPE-PEG2000, poly(d,l-lactide), 1,2-distearoyl-sn-glycero-3-phosphoethanolamine, methoxy (polyethylene glycol), 1,2-dilauroyl-sn-glycero-3-phosphocholine, collagen IV, polyethylene glycol-coating molecules; PLGA-b-PEG, biodegrad-able diblock poly (lactide-co-glycolide)-b-poly(ethylene glycol); PLGA-HDL:polymer poly (lactic-co-glycolic acid)-synthetic high-density lipoprotein; PS-PLGA-b-PEG, phosphatidylserine and PLGA-b-PEG self-assembling; sHDL, synthetic high-density lipoprotein; TC, total cholesterol; TG, Triglyceride.

### Nanoparticles loaded with LXR agonist GW3965

At the early stage of atherosclerosis, a large amount of collagen type IV (Col IV) are expressed in the plaques and are valuable targets for delivery of nanoparticles (Kamaly et al., 2016). With poly (D, L-lactide) (PLA)-based as the core, DSPE-mPEG1000/DLPC/Col IV-DSPE-PEG2000 as the lipid layer, these nanoparticles (Col IV-GW-NPs) show good binding to Col IV-targeted ligand linker. *In vitro*, Col IV-GW-NPs loaded with GW3965 upregulate LXR target genes and downregulate proinflammatory mediators in macrophages. *In vivo*, these nanoparticles can successfully reach the atherosclerosis lesion site. Compared with untargeted nanoparticles loaded with GW3965, the curative effect of Col IV-GW-NPs enhance approximately 18% in the LDLR^−/−^ mice. Moreover, these Col IV-GW-NPs do not induce fatty liver or hyperlipidemia during treatment (Yu et al., 2017).

Poly(lactide-co-glycolide)-b-poly (ethylene glycol) copolymers has high biocompatible and good biodegradable and can be prepared by self-assembly. These copolymers have been widely used as effective nanocarriers for small and macromolecular drugs (Farokhzad, 2008). These nanocarriers loaded with GW3965 (10 mg/kg) reduce atherosclerotic plaque formation by 50% in LDLR^−/−^ mice after retro-orbital injection for 2 weeks (three times weekly) (Zhang et al., 2015). Furthermore, the surface of these nanocarriers can be further modified by phosphatidylserine, which is a phagocytic target for phagocytes, such as macrophages (Fadok et al., 1992). In LDLR^*−/−*^ mice, these modified nanocarriers induce a 40% macrophage reduction in atherosclerotic plaques. Importantly, these nanocarriers loaded with GW3965 also reduce inflammation and have no negative impact on either plasma or liver compared with free GW3965 (Zhang et al., 2015). Sanchez-Gaytan et al. developed a hybrid polymer/HDL nanocarriers with lipid/apolipoprotein as the shell and poly (lactic-co-glycolic acid) as the core. Compared to normal poly (lactic-co-glycolic acid) nanocarriers, these new type nanocarriers not only have a slower release characteristic but also have the characteristics of natural HDL, such as preferential uptake by macrophages and good ability to transport cholesterol out of atherosclerotic plaque. In apoE^*−/−*^ mice, these hybrid polymer/HDL nanocarriers can successfully target atherosclerotic plaques and preferentially accumulate in plaque macrophages (Sanchez-Gaytan et al., 2015). These biomimetic nanocarriers provide a novel method for delivery of LXR agonists to macrophages in atherosclerosis.

### Nanoparticles loaded with LXR agonist T0901317

Among the drug loading nanocarriers, polyamidoamine dendrimer provides unique advantages for development of multifunctional dendritic nanoparticles (DNPs). Polyamidoamine dendrimers have more than 120 terminal amino groups in generation 5.0, which can be surface modified with high buffer capacity, and can achieve a unique “proton sponge” effect for endosome escape. In addition, PEGylation of DNPs can reduce positive charge on the surface, prolonging the blood circulation time of theses DNPs (Zahr et al., 2006; Lipka et al., 2010; Pelaz et al., 2015). Interestingly, the mannose functionalized dendrimer nanoparticles loaded with T0901317 can specifically target the MR expressed on macrophages. In LDLR^*−/−*^ mice, these intravenously injected mannose functionalized nanoparticles loaded with T0901319 (MDNP-T0901317) can successfully target macrophages in atherosclerotic plaques. Importantly, MDNP-T0901317 can be efficiently taken up by macrophages but not hepatocytes (He et al., 2018). This advantage is sure to reduce the side effects of T09011317 and other LXR agonists. A recent study reported a new nanoparticle, which uses mesoporous silica nanoparticles as carriers to generate nanoparticles that are simultaneously loaded with peroxisome proliferators-activated receptor alpha and LXRα agonists. These novel nanoparticles successfully reduce atherosclerotic plaque area in rats (Zhang W et al., 2022). It has been demonstrated that modification of nanoparticles with platelet membranes can significantly enhance the targeting property of nanocarriers (Wang H et al., 2020). Compared with uncoated nanoparticles, platelet membrane-coated nanoparticles can avoid complement activation, providing these nanoparticles with immune escape ability and making it possible for nanoparticles position in deep locations (Choi et al., 2020). This provides new ideas for the treatment of atherosclerosis using nanocarriers.

### Liposomes loaded with LXR agonists

Modification of liposomes with cyclic peptide Lyp-1 can make these liposomes be recognized by p32 receptors that are expressed on foam cells. Compared with untargeted liposomes, the accumulation of GW3965-Lyp-1 liposomes in foam cells of atherosclerotic plaque increases by 1.7 folds. In male LDLR^*−/−*^ mice, these modified liposomes reduce the number of macrophages in atherosclerotic plaques by 2 folds and increase the percentage of collagen by 3 folds. It is noteworthy that targeted liposomes loaded with GW3965-Lyp-1 can not only reduce atherosclerotic plaque formation but also stabilize atherosclerosis and reduce the side effects of LXR agonist. Furthermore, Lyp-1 is also used to functionalize other nanocarriers for targeting macrophages, such as heat shock protein cage designed by Uchida (Uchida et al., 2011). In another study using apoE^*−/−*^ mice, liposomes loaded with GW3965-Lyp-1 has a high retention rate in aortic lesions (3 h) and shows a significant therapeutic effect at a relatively low dose of GW3965 (≈6.5 mg kg^-1^) without inducing high serum TG levels (Seo et al., 2014).

### Nanohydrogels loaded with LXR agonists

Supramolecular hydrogels of short peptides have been widely studied due to their beneficial characteristics (Liu et al., 2015). D-glycine–phenylalanine–phenylalanine–tyrosine tetrapeptide (D-Nap-GFFY) modified with naphthyl acetic acid is not easy to be degraded by endogenous proteases due to the presence of amino acids in D-configuration. More importantly, these D-Nap-GFFY can successfully target macrophages. D-Nap-GFFY nanofiber hydrogels loaded with T0901317 are reported to inhibit atherosclerosis as that of oral T0901317 and do not induce hepatic lipogenesis. More importantly, D-Nap-GFFY-T0901317 hydrogels are found to reverse advanced atherosclerotic lesions (Ma et al., 2021).

### Synthetic HDL particles loaded with LXR agonists

HDL, an endogenous lipidic nanoparticles with a diameter ranging from 7 nm to 13 nm in size, is mainly composed of phospholipids and apoA-I. HDL can transfer cholesterol from lipid-laden plaque macrophages to the liver through the process of RCT. As preparation of ApoA-I from human plasma is time-consuming, various genetic variants of ApoA-I (Terasaka et al., 2003; Nicholls et al., 2018a) or recombinant ApoA-I (Tardif et al., 2014; Nicholls et al., 2018b) have been used to replace human plasma ApoA-I to obtain reconstituted HDL or synthetic HDL (sHDL) particles with similar arteriosclerosis protective effects as endogenous HDL. sHDL can effectively target atherosclerotic plaques and has been clinically proved to be a cholesterol receptor with good safety. (Jayaraman et al., 2012). sHDL particles have therefore been widely studied as nanocarriers to deliver therapeutics or imaging agents for atherosclerosis management (Lameijer et al., 2018; Binderup et al., 2019). These sHDL nanoparticles (10.5 ± 0.1 nm) loaded with LXR agonist T0901317 shows a good therapeutic effect with fewer side effects due to the low dosage of T0901317. In apoE^*−/−*^ mice fed a high fructose, high-fat diet for 6 weeks, these nanoparticles reduce aortic plaque areas by 15.0% (Yuan et al., 2021). In another study, phospholipid recombinant ApoA-I peptide (22A)-derived sHDL-nanoparticles loaded with T0901317 reduce the aortic root plaque area by 40.8% without induce side effects as that of free T0901317 (Guo T et al., 2018). HDL-like nanoparticles can also be prepared using full-length apoA-I protein, phospholipids, and polymers. The diameter of these HDL-like particles is about 30 nm, which is slightly greater than that of endogenous HDL (about 10–14 nm). In apoE^*−/−*^ mice, GW3965-laden HDL particles accumulate in aortic plaques and exhibit local anti-inflammatory effects (Tang et al., 2016). However, apoA-I is a large protein (human apoA-I has 243 amino acid residues), which makes it difficult and expensive to synthesize high-quality HDL particles for intravenous administration.

The advantages and disadvantages of these nanocarriers are listed in Table 2. For example, type IV collagen exposed in atherosclerotic plaques is a target, and PLA-DSPE NPs modified with type IV collagen have excellent physical stability and biocompatibility, but the preparation steps are cumbersome (Yu et al., 2017). Although PLGA-b-PEG NPs has good biodegradability and biocompatibility, the entrapment and loading efficiency are not high (Zhang et al., 2015). Macrophage TIM-4 receptors can recognize phosphatidylserine, and phosphatidylserine-modified PLGA-b-PEG NPs promote macrophages shift to an anti-inflammatory mode (Zhang et al., 2015). Mannose receptors are abundantly expressed on the surface of macrophages, and DNP NPs modified by mannose improve buffering capacity and prolong blood circulation time, but the preparation steps need to be simplified (He et al., 2018). Foam cell P32 receptors recognize Lyp-1, and liposomes modified by Lyp-1 can significantly penetrate atherosclerotic plaques, but P32 receptors are not only expressed on macrophages (Benne et al., 2020). Macrophage class A scavenger receptors recognize D-Nap-GFFY, and D-Nap-GFFY-modified nanohydrogels are highly stable *in vivo*. Although a small number of theses nanocarriers can be degraded by extracellular proteinase K, this type of nanocarriers has an attractive application in future (Ma et al., 2021). Furthermore, ApoA-I(22A)-sHDL NPs are well tolerated and have a long mean half-life and PLGA-HDL NPs have controlled release properties. However, they are expensive for preparation at present (Tang et al., 2016; Guo T et al., 2018; Yuan et al., 2021).

### Comparisons with other studies and what does the current work add to the existing knowledge

A recent article reviewed the application of nanoparticles for diagnostic imaging of CVD. They also discussed the applications of nanocarriers for therapy of hypertension, myocardial infarction, stroke, cardiomyopathy as well as atherosclerosis (Soumya and Raghu, 2023). Another review in 2022 described macrophage-targeted nanomedicines for diagnosis and treatment of atherosclerosis. The authors carefully reviewed nanoparticles for diagnostic imaging and discussed how rationally designed nanomaterials can efficiently carry and delivery therapeutics to macrophages (Chen et al., 2022). However, the above two review articles only mentioned a small portion of LXR agonists, which have a great potential application for therapy of atherosclerosis after successfully eliminating their side effects in the liver.

As described above, LXR expressed by macrophages are an excellent target for treatment of atherosclerosis. Activation of LXR not only inhibits macrophage-mediated inflammation but also promotes RCT, showing a good anti-atherosclerotic potential. However, LXR agonist-induced side effects, especially in the liver, have greatly limited the potential usage of these drugs. Targeted drug delivery system is designed to release drugs at the desired site, which can improve efficacy and reduce side effects. In this article, we provide useful information about the role of macrophages in atherosclerosis, the actions of LXR agonists (why they can induce side effects), how nanocarriers are designed and recognized by the receptors that are located on the surface of macrophages, and the advantages and disadvantages of these reported nanocarriers. This review will greatly enhance the research interest in macrophage-targeted therapy of atherosclerosis by LXR agonists as well as other drugs. Of importance, this review may promote the development of novel nanocarriers with greater targeting property and therapeutic effects, thereby promoting the potential clinical application of LXR agonists.

## Conclusion and future directions

Atherosclerosis therapy is still a great challenge for our humans. Macrophages play pivotal roles in the onset and development of atherosclerotic CVD. LXR play a unique role in lipid homeostasis and inflammation, which has attracted much attention in the field of developing therapeutic agents for atherosclerosis. However, until now no LXR agonists have not been approved as treatment for atherosclerosis which are partially due to their adverse effects including hepatic lipogenesis, steatosis and hyperlipidemia. Interestingly, macrophages express various receptors which can be used for targeted drug delivery. Present data clearly demonstrate that macrophage-targeted nanocarriers loaded with LXR agonists can successfully retard the progress of atherosclerosis and significantly reduce the side effects of these agonists. The success of nanocarriers in preclinical studies of arteriosclerosis and their application in human cancer treatment indicate that macrophage-targeted drug delivery system can be used in the diagnosis and treatment of patients with CVD in future.

However, there are some questions need to be addressed before these macrophage-targeted nanocarriers loaded with LXR agonists: 1) several kinds of nanocarriers have been studies by different groups, which kind of nanocarrier is the best for targeting macrophage and has the best therapeutic effect need to be further investigated by comparation studies; 2) distinct nanocarriers may have different drug release rate, pharmacokinetic studies are needed to figure out which kind of nanodrug delivery system is suitable for macrophage-targeted therapy of atherosclerosis; 3) there are several subtypes of macrophages in atherosclerotic plaques, whether nanocarriers can target special subtypes of macrophages and promote their shift (such as from M1 to M2) are an interesting problem needs to be figured out in future; 4) as patients with atherosclerotic CVD need a long-term intervention, the safety of the selected nanocarriers loaded with LXR agonists need to be investigated in animal experiments that are designed for more than 6 months’ treatment; 5) presently, the preparation of these nanocarriers, especially those biomimetic nanoparticles (such as synthetic HDL), is still a great challenge for their large scale production; 6) most researches rely on the mouse models, whose atherosclerotic plaques are different from our humans; 7) the last but not the least, the development of selective ligands for each LXR isoform may reduce the side effects of LXR agonist.
